# A New Class of Scandium Carbide Nanosheet

**DOI:** 10.1038/s41598-019-52882-3

**Published:** 2019-11-12

**Authors:** Jing Wang, Tian-Tian Liu, Chen-Ling Li, Ying Liu

**Affiliations:** 1 Department of Physics and Hebei Advanced Thin Film Laboratory, Hebei Normal University, Shijiazhuang, 050024 Hebei China; 2 National Key Laboratory for Materials Simulation and Design, Beijing, 100083 China

**Keywords:** Structural properties, Two-dimensional materials

## Abstract

A new class of two-dimensional scandium carbide nanosheet has been identified by using first-principles density functional theory. It has a primitive cell of Sc_3_C_10_, in which there are two pentagonal carbon rings surrounded by one scandium octagon. Being as the precussor of Volleyballene Sc_20_C_60_ and ScC nanotubes, the Sc_3_C_10_ nanosheet is exceptionally stable. By rolling up this Sc_3_C_10_ sheet, a series of stable ScC nanotubes have been obtained. All the nanotubes studied have been found to be metallic. Furthermore, the hydrogen storage capacity of the ScC nanotubes has been explored. The calculated results show that one unit of the (0,3) ScC nanotube can adsorb a maximum of 51 hydrogen molecules, reaching up to a 6.25 wt% hydrogen gravimetric density with an average binding energy of 0.23 eV/H_2_.

## Introduction

As two major types of two-dimensional (2D) materials, graphene and transition metal dichalcogenides (TMDCs), have been the object of intense investigations as potential materials for future nanoelectronics applications^1–6^. Developments in the field such as a field effect transistor require a moderate band gap, a reasonable carrier mobility, and excellent electrode-channel contacts^2,7^. As for graphene, it possesses remarkable electronic and mechanical properties, but the lack of a native band gap severely limits its applications in nanotransistors^8^. Recent great effort has been directed toward opening a band gap in various graphene-based nanosystems. Nonetheless the devices designed all have a low “on-off” current ratio^5^. For 2D TMDCs, the monolayer molybdenum disulfide (MoS_2_) does have a direct band gap (~1.8 eV^3^), but a carrier mobility of only about 200 cm^2^V^−1^s^−1^ ^6^, which is not sufficiently high for many applications^9^.

Recently, a new kind of 2D transition metal carbides, nitrides, and carbonitrides (MXenes) and their parent MAX phases (M = early transition metals, A = IIIA or IVA elements, X = carbon or nitrogen) have rendered them promising applications, such as energy storage^10,11^, water purification^12^, electromagnetic interference shielding^13^, and sensors^14^. Specifically, scandium-carbon systems have been found to have numerous phases, ScC, ScC_2_, Sc_2_C_3_, Sc_3_C_4_, Sc_4_C_3_, and Sc_13_C_10_. Among them, ScC, ScC_2_, Sc_2_C_3_, Sc_4_C_3_, and Sc_13_C_10_ are all cubic phases^15–17^, while Sc_3_C_4_ has been reported to be a tetragonal phase^18^. Herein, we propose a novel 2D scandium carbide, referred to below as Sc_3_C_10_ sheet. It possess robust stability and excellent structural and physical properties. In addition, it can be viewed as a precursor of Volleyballene Sc_20_C_60_^19–22^, as well as the ScC nanotubes.

Over the last several years, carbon-based nanomaterials, including carbon nanotube^23^, graphene^24^, and fullerene^25^ have been widely studied for the H_2_ storage applications due to their low weight and high specific surface area. However, the adsorption of H_2_ molecule is dominated by weak Van der Waals force, and only a small amount can be stored under ambient conditions. A possible way to enhance the interaction is by importing heteroatoms to synthesis novel carbon-based materials with large surface areas and pores^26–29^. BN nanotubes have been tested to be a better hydrogen storage medium than pure carbon nanotubes^28,29^. In this way, the hydrogen storage of this novel ScC nanotubes has been studied. It has been found that one unit of the (0,3) ScC nanotube can adsorb 51 hydrogen molecules and the hydrogen gravimetric density can reach up to 6.25 wt%.

## Results and Discussion

**Figure 1:**
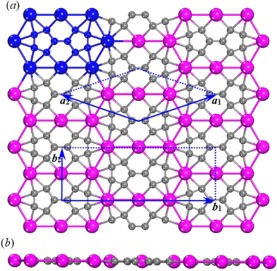
The configuration of the Sc_3_C_10_ naosheet, (**a**) top view and (**b**) side views. The large and small balls represent Sc and C atoms, respectively. The blue balls at the top left corner of (**a**) show the basic Sc_8_C_10_ subunit. The directed lines (***a***_**1**_, ***a***_**2**_ and ***b***_**1**_, ***b***_**2**_) represent the lattice vectors as described in the text.

Figure 1*a* shows the configuration of the most stable Sc_3_C_10_ sheet obtained in the structural search. The primitive cell contains 3 scandium atoms and 10 carbons with the chemical formula of Sc_3_C_10_. The lattice parameters are ***a***_**1**_ = ***a***_**2**_ = 8.855 Å and α = 142°, respectively. A unit cell (***b***_**1**_, ***b***_**2**_), twice the size of the primitive cell, is also given in Fig. 1*a*. In the Sc_3_C_10_ nanosheet, there is a basic structure, the Sc_8_C_10_ subunit, highlighted in the top left corner of Fig. 1*a*. In the Sc_8_C_10_ subunit, there are two carbon pentagons (C-pentagon) and one scandium octagon (Sc-octagon). It may be seen that each group of two C-pentagons is surrounded by one Sc-octagon, as the case of Sc_20_C_60_ Volleyballene^19^.

This new scandium carbide sheet may thus be viewed as consisting of Sc_8_C_10_ subunits set in a crisscross pattern. The average Sc-Sc bond length is 3.340 Å with two distinct Sc-Sc bond lengths: 3.351 Å along the horizontal direction (***b***_**1**_ in Fig. 1) and 3.328 Å for the other cases. For the C-pentagons, there are three C-C double bonds (1.428 Å), two C-C single bonds (1.466 Å), and one C-C bond of 1.437 Å connecting the two C-pentagons. Thus, the average C-C bond is 1.443 Å. For the Sc-C bond, the average value is 2.299 Å.

**Figure 2:**
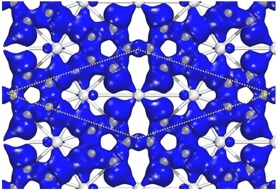
The deformation electron density of the Sc_3_C_10_ nanosheet. The iso-value of is set to 0.01 *e*/Å^3^.

**Figure 3:**
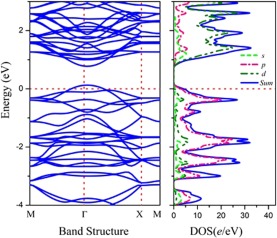
The band structure and partial density of states of the Sc_3_C_10_ sheet.

The stability of the Sc_3_C_10_ nanosheet was studied by analyzing the bond characteristics, and confirmed using *ab initio* molecular dynamics simulations. Figure 2 shows the deformation electron density, which reveals electron transfer from Sc atoms to carbons. Mülliken population analysis shows a charge transfer of ~0.6*e* for one Sc atom, mainly from Sc 3*d* state. On C atoms, it has obvious *sp*^2^-like hybridization. For Sc atoms, there are obvious *d* orbital characteristics. The Sc atom in the middle of the primitive cell bonds, through its *d* orbital, with the neighboring carbons. For the remaining two Sc atoms of the primitive cell, each Sc interacts with the two C atoms which are more centrally located than are the other six carbons. Close examination of the partial density of states (PDOS), as shown in Fig. 3, further confirms the hybrid characteristics between Sc *d* orbitals and C *s*-*p* orbitals. This is of great importance in stabilizing the planar Sc_3_C_10_ nanosheet.

Next, *ab initio* molecular dynamics simulations with an NVE ensemble were carried out with a time step of 1.0 *fs*. Here, a relatively large 2 × 2 supercell was used. The calculated results indicated that the Sc_3_C_10_ sheet retained its original topological structure and was not disrupted over a 5.0 *ps* dynamic simulation at a ~801 K effective temperature (*also see* Section I of the Supplementary Information). The snapshots of the geometries at the end of 5 *ps* simulations were given in Section I of the Supplementary Information. All the results indicate that the Sc_3_C_10_ naosheet has good thermodynamic stability. Finally, some typical variants of the Sc_3_C_10_ monolayer, consisting of the bilayer, trilayer, and bulk forms, were simulated at the same theoretical level and the calculated results were listed in Section II of the Supplementary Information.

Furthermore, the mechanical property and the electric structure have been analysed at the GGA/PBE level. It is found that the elastic constants of Sc_3_C_10_ sheet are 83.34, 70.27, and 23.71 N/m for C_11_, C_22_, and C_12_, respectively. According to the the equations of 2D system^30^, the Young’s modulus is obtained and the results are Y_[10]_ = 75.34 and Y_[01]_ = 63.54 N/m. The analysis of band structure (*see* Fig. 3) shows a direct band gap ~0.62 eV for the Sc_3_C_10_ nanosheet.

Just as graphene is the precursor of carbon nanotubes, a series of ScC nanotubes with different diameters and chiralities could be constructed based on the Sc_3_C_10_ nanosheet. We first specify how to describe these ScC nanotubes. Due to the low symmetry of this Sc_3_C_10_ nanosheet, it seems not appropriate to classify the ScC nanotubes by using the primary vectors (***a***_**1**_, ***a***_**2**_) of the orthorhombic lattice. The lattice vectors of the rectangular lattice, ***b***_**1**_ and ***b***_**2**_ (as shown in Fig. 1*a*), seem to be more appropriate and convenient for labelling ScC nanotubes with integer multiples of the rectangular lattice vectors. Here we considered two kinds of tubes: (*p*, 0) and (0, *q*), where *p* and *q* are integers. The *p****b***_**1**_ and *q****b***_**2**_ represent the vectors of a strip which will be rolled up to a nanotube.

**Figure 4:**
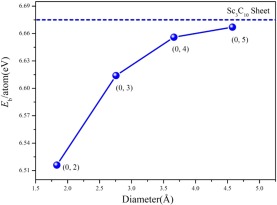
The binding energy per atom of ScC nanotubes *vs* the diameter. The labels describe the nanotubes in terms of the rectangular lattice vectors of the Sc_3_C_10_ nanosheet as outlined in the text.

**Figure 5:**
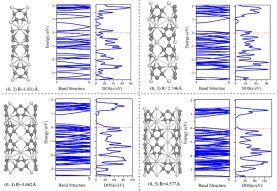
The band structures and densities of states for (0, *q*) nanotubes with *q* = 2, 3, 4, 5, as well as the schematics of corresponding ScC nanotubes.

Calculations were performed on these tubes. After geometry optimization, it was found that the (*p*, 0) tubes with *p* = 1, 2, 3 had all collapsed. Only the (0, *q*) nanotubes with *q* = 2, 3, 4, 5 were stable. For these (0, *q*) nanotubes, the diameters are in the range 1.83–4.53 Å, and the stabilities and electronic properties have been explored. Figure 4 lists the binding energy per atom of the (0, *q*) nanotubes *vs* the diameter. It can be seen that with the increase of diameter the binding energy approaches the value of the corresponding Sc_3_C_10_ nanosheet. The (0, *q*) ScC nanotubes of large diameter have relatively high stability. Analysis of the electronic structures of the (0, *q*) ScC nanotubes indicates that all four (0, *q*) tubes rolled from the Sc_3_C_10_ nanosheet are metallic. The band structures and densities of states (DOS) of the (0, *q*) ScC nanotubes are shown in Fig. 5. Close examination of the band structures indicates that the (0, 2) nanotube is different from the other three examined. For the latter cases, all of the (0, 3), (0, 4), and (0, 5) nanotubes exhibit a gap slightly above the fermi level. All three band gaps are direct band gaps at the *Г*-point and the gap sizes increase as the diameter increases. The band gaps are ~0.60, 0.64, and 0.71 eV for the (0, 3), (0, 4), and (0, 5) nanotubes, respectively. The band structure of the (0, 2) tube, on the other hand, shows several bands in the vicinity of the fermi level, which ensures a large carrier density. The above results indicate that these ScC nanotubes may have potential applications in metallic connections of electronic devices.

Then, the hydrogen adsorption of (0,3) ScC nanotube was discussed. As we known that the van der Waals (vdW) interactions are important for the formation and stability of molecules. The hybrid semiempirical dispersion-correction approach of the Tkatchenko-Scheffler (TS) scheme^31^ was employed during the optimization.

**Figure 6:**
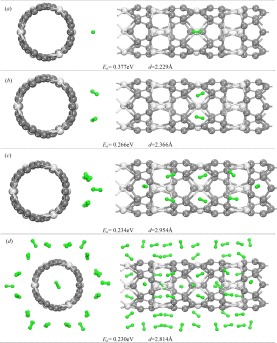
Optimized structure of (0,3) ScC nanotube with the adsorbed hydrogen molecules. (**a,b**) for the case of one Sc atoms, (**c**) for the case of the group of three Sc atoms, and (**d**) for the case of one unit cell. Two columns are viewing from different angles. Below are *E*_*a*_ and *d*.

We first considered the interaction between Sc atom and hydrogen molecules, and the Sc lying in the middle of the unit of the (0,3) ScC nanotube was selected. Figure 6(a,b) shows the configurations of H_2_ adsorption on the selected Sc atom, as well as the average adsorption energy of hydrogen molecule (*E*_*a*_) and the average distance between hydrogen molecule and Sc atom (*d*). The first hydrogen molecule tend to the site right above the Sc atom and lies parallel to the axis of the tube. The adsorption energy of the first adsorbed H_2_ is 0.377 eV lying in the range 0.1–0.6 eV, which was a suggested criterion for the H_2_ storage medium. The distance of the hydrogen molecule to Sc atom is 2.229 Å indicating a strong van der Waals interaction between hydrogen molecule and the ScC nanotube. When adsorbed two hydrogen molecules, the H_2_ molecules prefer to form a line vertical to the axis of the ScC nanotube. It has only a small change for the distance between hydrogen and Sc atom (2.325 and 2.406 Å). For the second hydrogen molecule, the adsorption energy is 0.155 eV smaller than that of the first one. When the third H_2_ were added, the energy minimization indicated that the third H_2_ molecule prefers to the neighbor Sc atoms. It may due to the limited Sc-Sc distance (3.356 Å) of the (0, 3) ScC nanotube.

Thus, we take the neighboring three Sc atoms, which lie on a line parallel to the axis of the tube, as a group to consider the situation of their hydrogen adsorption. It is found that the three Sc atoms can adsorb eight H_2_ molecules in maximum. Besides each Sc atom can adsorb two H_2_, just as the case of one Sc atom, one more H_2_ can adsorb by the side Sc atoms arranging along the axis of the tube, as shown in Fig. 6(c). The average distance, *d*, becomes large. It is 2.954 Å with the *E*_*a*_ equals to 0.234 eV/H_2_. For (0,3) ScC nanotube, a total of 51 H_2_ molecules were adsorbed onto one unit, with three H_2_ molecules in the middle of the tube (*see* Fig. 6d). Then a hydrogen storage capacity of 6.250 wt% is obtained for the (0,3) ScC nanotube, which is in excess of 6 wt%, the U. S. Department of Energy target. The average distance and the average adsorption energy are 2.814 Å and 0.230 eV/H_2_, respectively.

## Conclusions

In conclusion, our first-principles investigations have proposed a stable Sc_3_C_10_ nanosheet using both static and dynamic *ab initio* calculations. The new scandium carbide nanosheet may be viewed as consisting of Sc_8_C_10_ units arranged in a crisscross pattern. Hybridization between Sc *d* orbitals and C *s*-*p* orbitals is essential for stabilizing the Sc_3_C_10_ nanosheet. Furthermore, all the stable ScC nanotubes rolled from this Sc_3_C_10_ nanosheet were found to be metallic within the scope of the approximations used in our research. The hydrogen storage property of ScC nanotube has also been explored. For one unit of (0,3) ScC nanotube, the number of adsorbed hydrogen molecules can reach up to 51, corresponding to a 6.25 wt% hydrogen uptake with *Ec* = 0.230 eV/H_2_. All these prediction are expected to motivate experimental efforts in view of the fundamental value and potential applications of ScC nanostructures.

## Methods

Our calculations were performed within the framework of spin-polarized density functional theory (DFT) with the generalized gradient approximation (GGA) using the exchange-correlation potential described by Perdue-Burke-Ernzerhof (PBE)^32^. As described previously^33–35^, the calculations were carried out with unrestricted symmetry using a double-numerical polarized (DNP) basis set^36^. For Sc atom, the DFT semi-core pseudopotentials (DSPP)^37^ was used and for C atom all electrons were included in the calculation. All structures were fully relaxed, and geometric optimizations were performed with convergence thresholds of 10^−5^ hartree (Ha) for the energy, 2 × 10^−3^ Ha/Å for forces, and 5 × 10^−3^ Å for the atomic displacements. For the ScC nanotube calculations, the vacuum spaces between tubes were made larger than 10 Å to avoid interactions between the tubes.

## Supplementary information

Supplementary Information
