# Supplementary material for: A New Class of Scandium Carbide Nanosheet
Source: Sci Rep. 2019 Nov 12;9:16624. doi: 10.1038/s41598-019-52882-3 (PMC6851380; doi:10.1038/s41598-019-52882-3)
Supplement: Supplementary file 1 — Supplementary Information [file 41598_2019_52882_MOESM1_ESM.docx]

Supplementary Information

A New Class of Scandium Carbide Nanosheet

Jing Wang,^1^ Tian-Tian Liu,^1^ Chen-Ling Li,^1^ and Ying Liu*^,1,2^

^1.^ Department of Physics and Hebei Advanced Thin Film Laboratory, Hebei Normal University, Shijiazhuang 050024, Hebei, China.

^2.^ National Key Laboratory for Materials Simulation and Design, Beijing 100083, China.

^*^*Correspondence and* *request for materials should be addressed to Y. Liu (yliu@ hebtu.edu.cn)*

^1^

**Section I．The molecular dynamic simulation of 2×2 supercell**

The *ab initio* molecular dynamic simulation with 2×2 supercell was further carried out for the Sc_3_C_10_ monolayer. In the calculations, seven diﬀerent initial temperatures of 300K, 500K, 700K, 1000K, 1200K, 1400K, and 1500K were used. Figure S1 lists the snapshots of the geometries at the end of 5 *ps* simulations. All the results show that the Sc_3_C_10_ monolayer can maintain its original topological structures at an initial temperature up to 1200K, but it will collapse at extremely high initial temperatures more than 1400K.

| 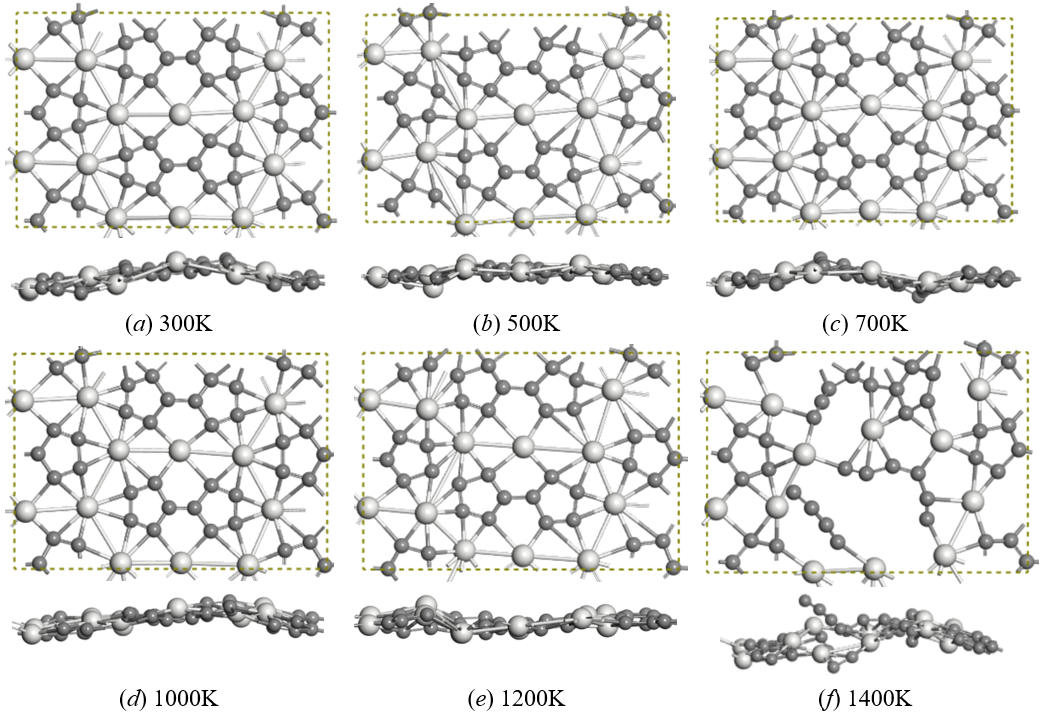 |
| --- |
| **Figure S1**. Snapshots for the equilibrium structures of Sc_3_C_10_ monolayer at the temperatures of (*a*) 300K, (*b*) 500K, (*c*) 700K, (*d*) 1000K, (*e*) 1200K, and (*f*) 1400K, at the end of 5*ps* *ab initio* dynamic simulations. |

As to the effective temperature, it was evaluated by averaging all the temperatures of each simulation step. Here, we take the initial temperatures of 1000K and 1200K for example. By counting and averaging the temperature values of each simulation step, the effective temperatures of 592.7K and 801.4K were obtained. Figure S2 lists the curves of temperature *vs* simulation time and the constant of motion *vs* simulation time during the simulations.

| 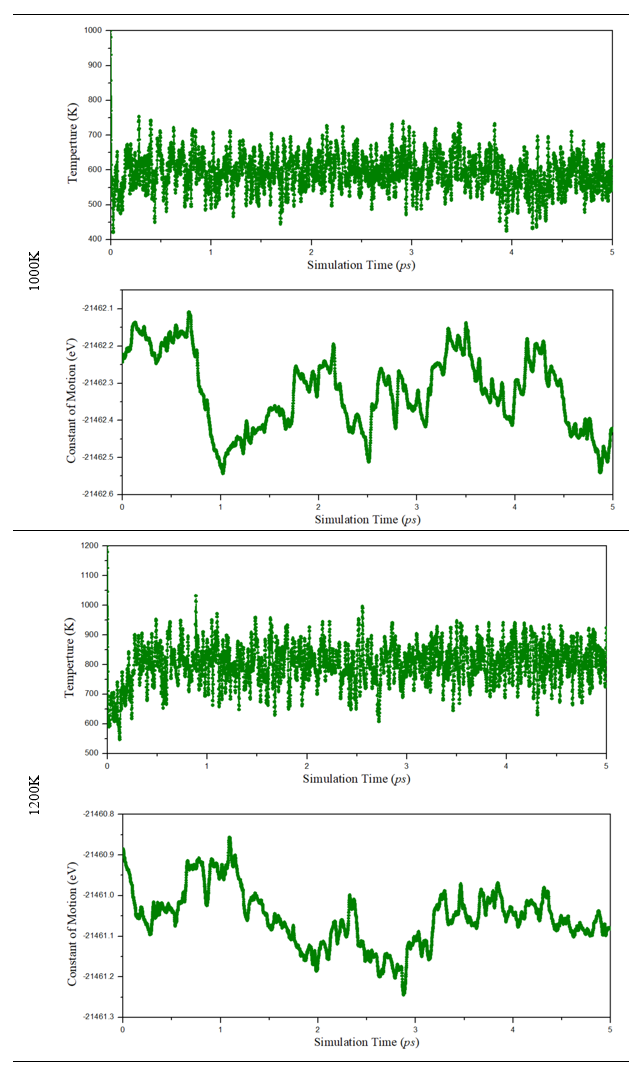 |
| --- |
| **Figure S2**. The curves of temperature *vs* simulation time and the constant of motion *vs* simulation time for the *ab initio* molecular dynamic simulations at initial temperatures of 1000K and 1200K. |

**Section II．The Sc_3_C_10_ bilayer, trilayer, and the bulk phase**

The Sc_3_C_10_ bilayer, trilayer, and the bulk phase with AA stacking were investigated at the GGA/PBE level. All the results, including configurations and electronic properties, were listed in Figure S3. From Figure S3, it can be seen that the Sc_3_C_10_ sheet can keep its 2D structure in the forms of bilayer and bulk phase, while there appeared a low distortion in the Sc_3_C_10_ trilayer. The band structure and partial density of states showed that the AA-stacked Sc_3_C_10_ bilayer, trilayer, and bulk phase are all metallic.

| 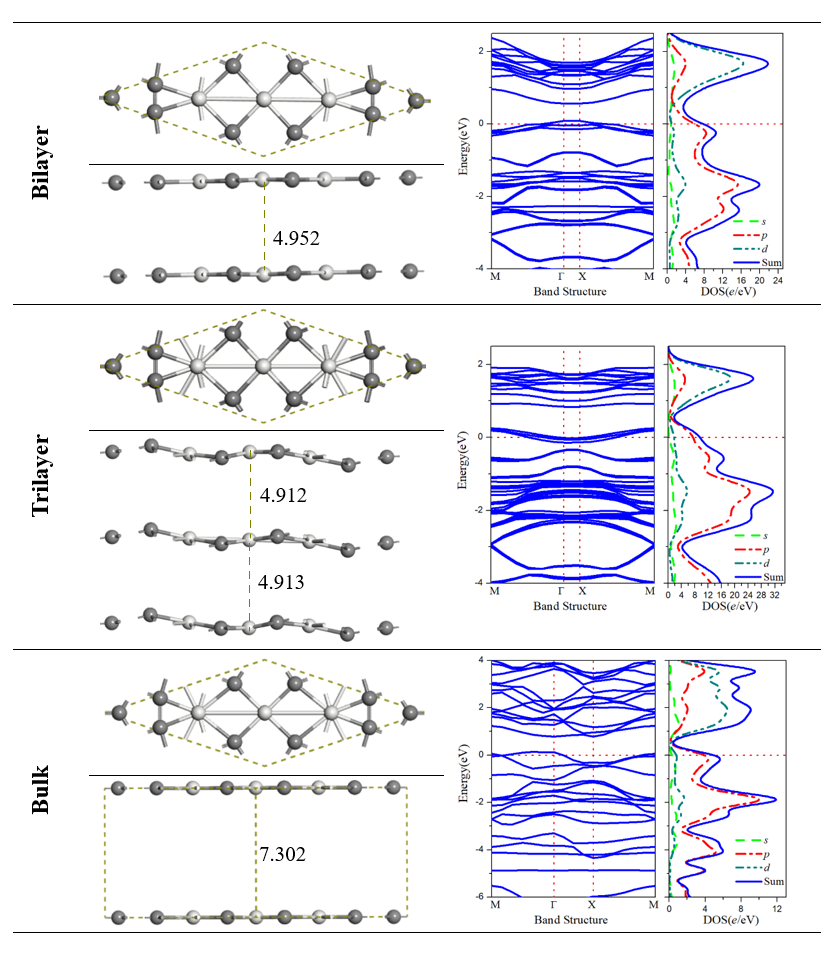 |
| --- |
| **Figure S3**. The conﬁgurations (top and side views), band structures, and the density of states (DOS) of AA-stacked Sc_3_C_10_ bilayer, trilayer, and the bulk phase. |

**Section III．The Adsorption of C and O atoms**

The absorption of carbon (C) and oxygen (O) atoms on the surface of Sc_3_C_10_ sheet were analyzed with a 2×1 supercell. Here, we have considered different adsorption sites including top of C/Sc, the bridge of C-C, Sc-C, Sc-Sc, and the middle of the C-pentagons. The energetically more favorable positions are determined as shown in Figure S3, as well as the first two low-lying isomers. It can be seen that both C and O atom prefer top cross site of the C-C and Sc-Sc.

| 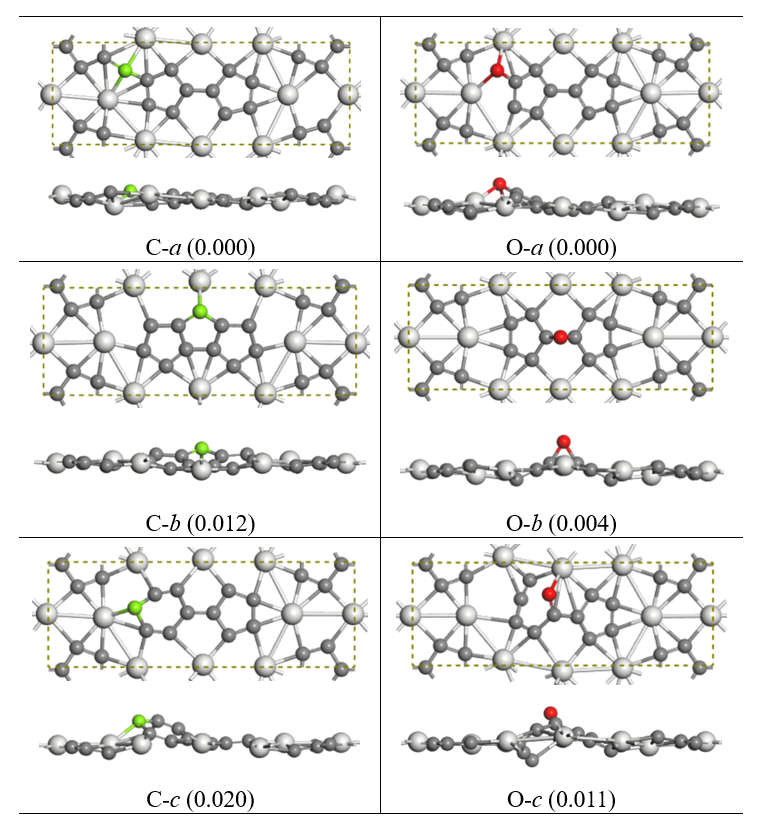 |
| --- |
| **Figure S4**. Top and side views of the lowest-energy conﬁgurations and the first two low-lying isomers for a C/O atom adsorbed on the Sc_3_C_10_ sheet. The numbers under the structures are the differences of the total energy (in eV) with respect to the lowest-energy structure. The green and red atoms represent carbon and oxygen atoms, respectively. |
